# Gemcitabine Functions Epigenetically by Inhibiting Repair Mediated DNA Demethylation

**DOI:** 10.1371/journal.pone.0014060

**Published:** 2010-11-19

**Authors:** Andrea Schäfer, Lars Schomacher, Guillermo Barreto, Gabi Döderlein, Christof Niehrs

**Affiliations:** 1 Division of Molecular Embryology, DKFZ-ZMBH Alliance, Deutsches Krebsforschungszentrum, Heidelberg, Germany; 2 Institut für Molekulare Biologie, Mainz, Germany; University of Barcelona, Spain

## Abstract

Gemcitabine is a cytotoxic cytidine analog, which is widely used in anti-cancer therapy. One mechanism by which gemcitabine acts is by inhibiting nucleotide excision repair (NER). Recently NER was implicated in Gadd45 mediated DNA demethylation and epigenetic gene activation. Here we analyzed the effect of gemcitabine on DNA demethylation. We find that gemcitabine inhibits specifically Gadd45a mediated reporter gene activation and DNA demethylation, similar to the topoisomerase I inhibitor camptothecin, which also inhibits NER. In contrast, base excision repair inhibitors had no effect on DNA demethylation. In *Xenopus* oocytes, gemcitabine inhibits DNA repair synthesis accompanying demethylation of *oct4*. In mammalian cells, gemcitabine induces DNA hypermethylation and silencing of *MLH1*. The results indicate that gemcitabine induces epigenetic gene silencing by inhibiting repair mediated DNA demethylation. Thus, gemcitabine can function epigenetically and provides a tool to manipulate DNA methylation.

## Introduction

Gemcitabine (2′deoxy-2′2′-difluorocytidine monohydrochloride, GEMZAR) is one of the most widely used anti-cancer drugs and is particularly effective against solid tumors [1]. Pioneering work from the Plunkett laboratory showed that gemcitabine is a pro-drug, which after intracellular uptake is metabolized to gemcitabine diphosphate and triphosphate, whose incorporation into DNA results in chain termination by inhibiting DNA polymerase activity [2], [3]. Unlike its analog cytosine arabinoside, which causes immediate termination of DNA polymerization, gemcitabine allows limited nucleotide polymerization by a process termed masked chain termination, which prevents exonucleases from excising the aberrant gemcitabine nucleotide [4], [5]. Incorporated gemcitabine can be recognized by p53 and DNA dependent protein kinase, which may induce apoptosis [6]. Gemcitabine also potently inhibits ribonucleotide reductase, resulting in a decrease of competing deoxyribonucleotide pools necessary for DNA synthesis [5], [7], [8]. Thus, gemcitabine inhibits DNA synthesis by at least two different modes. Gemcitabine can also induce increased ligase I levels [9].

Gemcitabine is frequently used in combination with cisplatin, which forms DNA adducts, that can be repaired by nucleotide excision repair (NER). The synergistic action of both drugs is thought to reside in an inhibitory effect of gemcitabine on the repair of the DNA lesions induced by cisplatin [10], [11], [12]. The current model is that gemcitabine inhibits DNA repair synthesis, which is an obligatory step in NER and thereby potentiates cisplatin effects.

Recently, we have implicated NER in the removal of 5′-methylcytosine (5mC) from DNA during active DNA demethylation [13]. In DNA of metazoa, 5mC is a common epigenetic mark associated with gene silencing, which can be reversed by active DNA demethylation. We showed that Growth Arrest and DNA Damage inducible protein 45 a (Gadd45a) is a key mediator of active DNA demethylation [13]. Gadd45a binds directly to and requires the activity of Xeroderma pigmentosum complementation group protein G (XPG), a 3′endonuclease of the NER complex. We therefore suggested a model where Gadd45a is targeted to specific sites of demethylation and recruits the DNA repair machinery. Methylated cytosines are then excised and replaced by unmethylated nucleotides [13].

Since gemcitabine inhibits NER, it was of interest if it also affects DNA methylation. Here we tested this possibility and find that gemcitabine inhibits specifically Gadd45a mediated reporter gene activation. Moreover, gemcitabine inhibits unscheduled DNA synthesis in methylated *oct4* plasmid in *Xenopus* oocytes. Finally, it induces hypermethylation and inhibits expression of *MLH1*. The results therefore indicate a new epigenetic mode of gemcitabine action.

## Results and Discussion

We first examined gemcitabine along with other cytotoxic drugs in a methylation sensitive reporter assay, where we monitored *Gadd45a*-mediated re-activation of an *in vitro* methylated – and hence silenced - Gal-responsive luciferase reporter plasmid [13]. The Gal4 reporter system is based on the ability of GAL4-Elk1 fusion protein to specifically bind and activate a Gal4 driven luciferase gene [14], [15]. Camptothecin and β-lapachone are inhibitors of topoisomerase I, an enzyme required during DNA repair [16]. Etoposide and merbarone are inhibitors of topoisomerase II, which is not involved in NER or base excision repair (BER) [17], [18].

**Figure 1:**
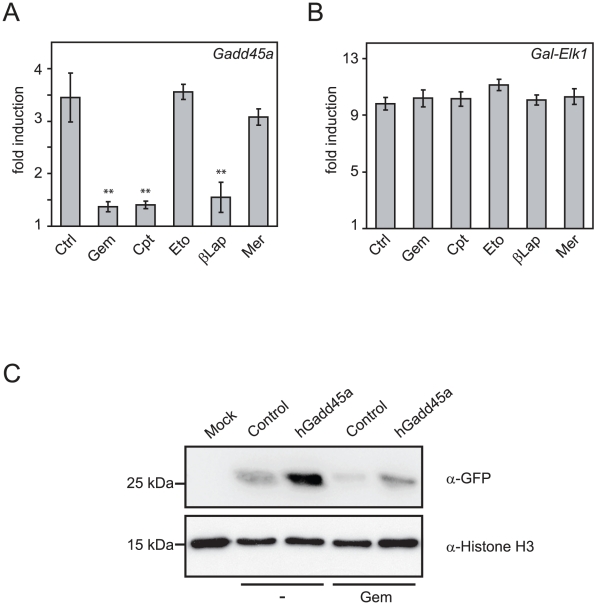
Gemcitabine inhibits Gadd45a mediated gene activation. (**A–B**) Luciferase reporter assays of HEK293T cells transiently transfected with HpaII *in vitro* methylated Gal-responsive reporter, together with either *Gadd45a* (A) or *Gal-Elk1* (B, specificity control). Cells were treated with DMSO (control, Ctrl), gemcitabine (Gem), camptothecin (Cpt), etoposide (Eto), β-lapachone (βLap), merbarone (Mer) as indicated. Shown is the fold activation by Gadd45a (A) or Gal-Elk1 (B) over control transfected cells. Error bars represent standard deviation. Significance was assessed via unpaired Student's t-test using the control sample as reference: **  = p<0.01. (**C**) Western blot analysis of EGFP expression. Whole cell extracts of HEK293T cells transiently transfected with *in vitro* methylated *pOctTK-EGFP* reporter with *Gadd45a* or *pBl-KS* (control), with or without gemcitabine treatment as indicated.

All three DNA repair inhibitors, gemcitabine, camptothecin and β-lapachone inhibited *Gadd45a*-mediated activation of the reporter (Fig. 1A). In contrast, the topoisomerase II inhibitors etoposide and merbarone had little effect. Importantly, activation of the same methylated reporter plasmid by the transcriptional activator *Gal-Elk1* (Fig. 1B) as well as activation of the cotransfected *Renilla* luciferase reporter plasmid used for normalization (not shown), were unaffected by the DNA repair inhibitors, ruling out unspecific inhibitory effects of these compounds on transcription and/or translation. Furthermore, an *in vitro* methylated *EGFP* reporter plasmid under the control of the *oct4* regulatory region fused to the thymidine kinase promoter was transcriptionally activated by Gadd45a as monitored by the re-expression of EGFP (Fig. 1C). This re-activation was also impaired by gemcitabine treatment.

**Figure 2:**
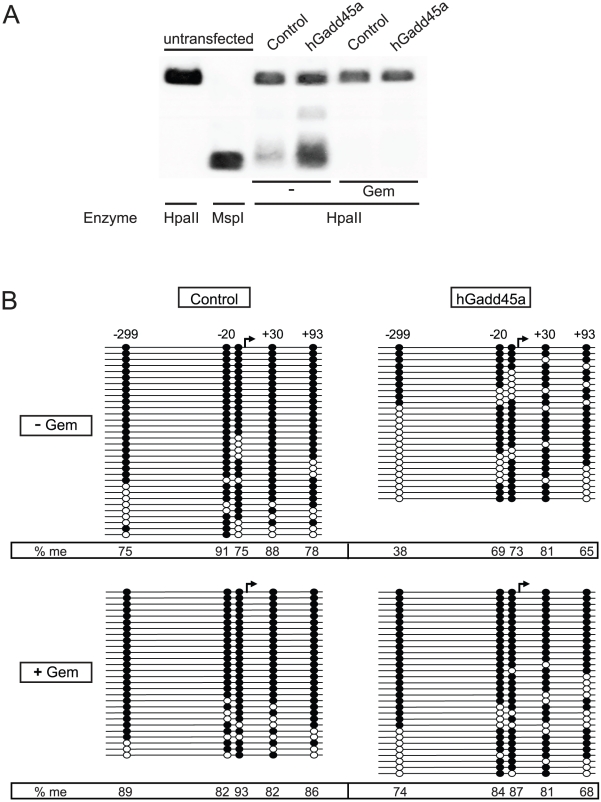
Gemcitabine impairs Gadd45a mediated demethylation. (**A**) Methylation-sensitive Southern blot. HpaII *in vitro* methylated plasmid *pOctTK-EGFP* was recovered from HEK293T cells after transient co-transfection with *Gadd45a* or *pBl-KS* (control), with 65 h gemcitabine treatment as indicated. Recovered plasmids were digested with the indicated restriction enzyme and the products analyzed by Southern blot using a *GFP* probe. (**B**) Bisulfite sequencing analysis of five HpaII sites within the *pOctTK* regulatory region upon transient transfection and treatment as in (A). White and black circles, unmethylated, methylated CpG, respectively. Arrow marks EGFP translation start site.

To directly test if this transcriptional repression by gemcitabine is indeed due to DNA hypermethylation, we monitored methylation levels using methylation sensitive Southern blotting. Untransfected *in vitro* methylated reporter plasmid was expectedly resistant to the methylation sensitive restriction enzyme HpaII, but digested by the methylation insensitive isoschizomer MspI (Fig. 2A). Following transfection, the reporter was mostly HpaII insensitive, while its co-transfection with Gadd45a induced HpaII sensitivity, indicating DNA demethylation. Treatment with gemcitabine impaired this demethylation.

To independently corroborate these results, we employed bisulfite sequencing. We first confirmed that the reporter was initially fully methylated (Fig. S1). Sequencing of the reporter recovered from transfected cells revealed, interestingly, some spontaneous demethylation. Gadd45a overexpression induced substantial demethylation of the EGFP reporter, most pronounced (two-fold) at the site -299 (Fig. 2B). Importantly, gemcitabine treatment reversed this effect resulting in methylation levels comparable to control without Gadd45, and also reduced endogenous demethylation. These results supports that gemcitabine inhibits Gadd45a mediated DNA demethylation. Furthermore, since endogenous demethylation is also gemcitabine sensitive this may involve endogenous Gadd45a and NER.

**Figure 3:**
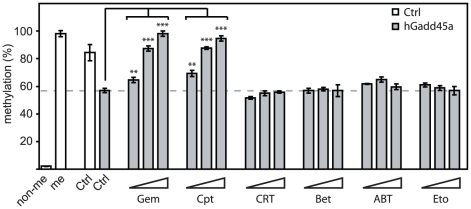
Gadd45a mediated DNA demethylation is unaffected by BER inhibitors. Methylation status of the HpaII site −299 (see Fig. 2B) in the *pOctTK-EGFP* regulatory region was assayed by methylation sensitive PCR 48 h after transient co-transfection with or without *hGadd45a*. Cells were treated with the topoisomerase II inhibitor etoposide (Eto), the NER inhibitors gemcitabine (Gem) and camptothecin (Cpt), or the BER inhibitors CRT 0044876 (CRT), betulinic acid (Bet) and ABT-888 (ABT) as indicated. Untransfected unmethylated (non-me) and HpaII *in vitro* methylated reporter plasmid (me) served as reference. Significance was assessed via unpaired Student's t-test using the untreated Gadd45a transfected sample as reference: *  = p<0.05; **  = p<0.01; ***  = p<0.001.

Besides NER, a base excision repair-based mechanism (BER) has been implicated in active DNA demethylation in mammalian cells [19], [20], [21]. Moreover, Gadd45a may also affect BER in addition to its effect on NER [19], [22]. Since BER also requires DNA synthesis, the question arose if gemcitabine may function as a BER inhibitor. We therefore tested bona fide BER inhibitors. CRT 0044876 (CRT) and betulinic acid (Bet) are inhibitors of AP Endonuclease I, [23] and DNA Polymerase β [24], respectively, both of which are key enzymes in BER. Furthermore, ABT-888 (ABT) blocks PARP-1, a sensor of single and double strand breaks during BER [25]. Remarkably, none of these compounds affected Gadd45a mediated demethylation of the *pOctTK-EGFP* reporter as assessed by methylation sensitive PCR (Fig. 3). This suggests that BER does not play a role in Gadd45a mediated demethylation, at least in this context. Under the same conditions, camptothecin (Cpt) as well as gemcitabine (Gem) blocked the Gadd45a induced DNA demethylation as well as endogenous demethylation, again supporting a NER model for Gadd45a demethylation.

DNA demethylation can theoretically also occur in a passive manner if the reporter plasmid is repetitively replicated. To experimentally rule out this scenario in our reporter system, we performed methylation sensitive PCR assaying the bacterial methylation state of the transfected plasmid (Fig. S2). A single ClaI recognition site in the backbone of *pOctTK-EGFP* is also target for bacterial Dam methylation. Bacterial Dam methylation blocks ClaI restriction at this site. During replication in eukaryotic cells, the bacterial methylation would be diluted if the plasmid was replicating and would gain ClaI sensitivity. While the reporter from *dam^−^* cells was sensitive to ClaI, the *pOctTK-EGFP* from *dam* ***^+^*** *E.coli* remained resistant to ClaI digest 65 h after transfection and thus was not replicated in the transfected cells. Hence, Gadd45 mediated demethylation is replication-independent and therefore active.

**Figure 4:**
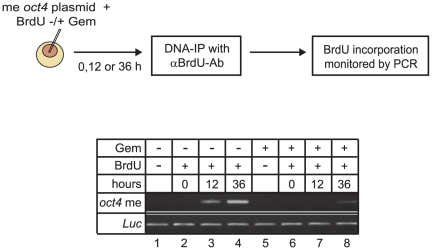
Gemcitabine inhibits unscheduled DNA synthesis of methylated DNA. Methylated *oct4* plasmid was injected with or without BrdU into *Xenopus* oocytes in presence or absence of gemcitabine (Gem) and recovered after incubation (see diagram). To control for equal loading, *in vitro* BrdU labeled luciferase plasmid (*Luc*) was added after oocyte lysis. PCR analysis of immunoprecipitated DNA using *oct4* or *Luc* specific primers was carried out.

We showed previously that *Gadd45a* is required for DNA demethylation of the *oct4* promoter in *Xenopus* oocytes. This demethylation is accompanied by unscheduled DNA repair synthesis, since Bromo-deoxyuridine (BrdU) is incorporated into methylated but not unmethylated *oct4* plasmid [13]. *Xenopus* oocytes are resting cells, and hence BrdU incorporation cannot be due to replication but rather be related to DNA repair processes. We therefore tested if this unscheduled DNA repair synthesis is sensitive to gemcitabine. BrdU was coinjected in oocytes with methylated *oct4* plasmid with or without gemcitabine. After 0, 12, or 36 h, plasmid DNA was immunoprecipitated with anti-BrdU antibodies and analyzed by PCR. The amount of PCR product obtained is a measure of BrdU incorporation and hence, DNA synthesis. In control samples, a progressive PCR product increase is observed with time (Fig. 4, lanes 2–4) [13]. Significantly, gemcitabine treatment almost completely abolished this BrdU incorporation (lanes 6–8). This result suggests that gemcitabine inhibits DNA repair synthesis associated with DNA demethylation. As a caveat, we cannot rule out the possibility that the reduced PCR product after BrdU immunoprecipitation is due to an inhibitory effect of Gemcitabine or its metabolites on Taq DNA polymerase. However, our data obtained by various methods (methylation-sensitive PCR, Southern blotting, bisulfite sequencing) argue that the effects of gemcitabine are rather due to inhibition of DNA repair.

**Figure 5:**
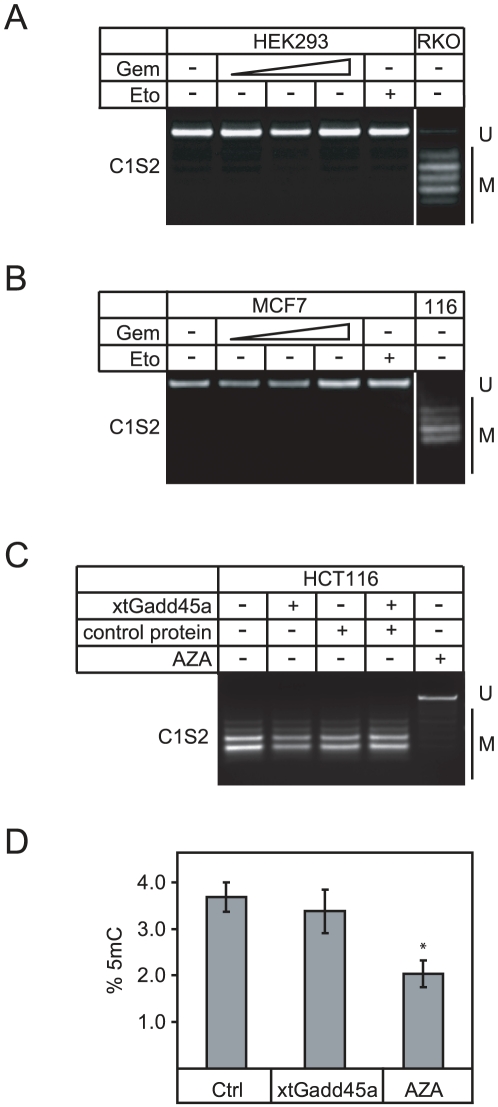
Gemcitabine does not affect global methylation levels. (**A–B**) Methylation of chromosome 1 satellite 2 (C1S2) in HEK293 (A) or MCF7 cells (B) was analyzed by combined bisulfite restriction analysis (COBRA). Note that neither gemcitabine (Gem, 33–134 nM) nor etoposide (Eto, 43 nM) treatment affect C1S2 methylation. C1S2 methylation of RKO cells (A, right) and HCT116 cells (116, B, right) serve as control for high C1S2 methylation. U, undigested (unmethylated) PCR amplicon; M, digested (methylated) restriction fragment. (**C**) COBRA analysis of C1S2 methylation in HCT116 cells as in (A, B). Cells were transfected with *X. tropicalis Gadd45a* (*xtGadd45a*) or treated with 5-aza-2′-deoxycytidine (AZA). U, undigested (unmethylated) PCR amplicon; M, digested (methylated) restriction fragment. (**D**) Capillary electrophoresis (CE) of DNA 5-methylcytosine (5mC). HCT116 cells were either treated with AZA or transiently transfected with *X. tropicalis Gadd45a* (xtGadd45a). After 48 h 5mC levels were determined by CE. Error bars represent standard deviation. Significance was assessed via Student's t-test using the untreated sample as reference: *  = p<0.05.

Next, we analyzed the effect of gemcitabine on methylation of endogenous loci and first examined global methylation levels. The bulk of 5mC in the genome is associated with telomeres and repetitive DNA, rather than transcribed genes. As cancer is often associated with global DNA hypomethylation, in particular hypomethylated chromosome 1 satellite 2 repetitive elements (C1S2) [26], [27], we analyzed the effect of gemcitabine on the methylation of these elements. Gemcitabine did not alter C1S2 methylation in HEK293 or MCF7 cells at any tested concentration or at any time point analyzed (18–42 h after treatment, data not shown) (Fig. 5A–B). This was surprising, since we previously reported that Gadd45a induces C1S2 demethylation and global hypomethylation. However, using improved experimental conditions we now found that Gadd45a overexpression does not induce significant C1S2 demethylation or global hypomethylation in HCT116 cells, unlike the demethylating drug 5-aza-2′-deoxycytidine (AZA) (Fig. 5C–D). Rather, the major demethylation effect of Gadd45 appears to be restricted to single copy genes ([19], [28], [29] and our unpublished data).

**Figure 6:**
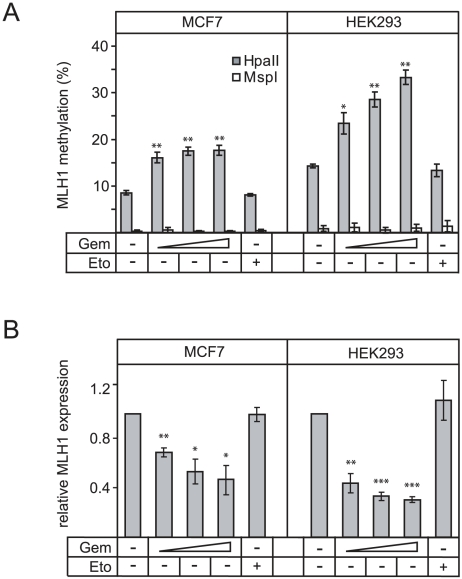
Gemcitabine induces hypermethylation and silencing of *MLH1*. (**A**) Methylation sensitive PCR analyzing *MLH1* promoter methylation state in MCF7 or HEK293 cells. Cells were treated as indicated with increasing concentrations of gemcitabine (Gem, 33–134 nM) or etoposide (Eto, 43 nM) as control. HpaII restriction is methylation sensitive and allows the quantification of the *MLH1* methylation state. As control, samples were also treated with the methylation insensitive isoschizomer MspI. Error bars represent the standard deviation of three biological replicates. Significance was assessed via unpaired Student's t-test using the untreated sample as reference: *  = p<0.05; **  = p<0.01. (**B**) Cells were treated as in (A). Relative expression of *MLH1* normalized to *GAPDH* was monitored by qPCR. Error bars represent the standard deviation of three biological replicates. Significance was assessed via unpaired Student's t-test using the untreated sample as reference: *  = p<0.05; **  = p<0.01; ***  = p<0.001.

We therefore analyzed the effect of gemcitabine on DNA methylation of an endogenous single-copy gene. The promoter of *MLH1* is a well studied methylation regulated gene which is kept partially unmethylated by Gadd45a [13]. Treating HEK293 and MCF7 cells with increasing amounts of gemcitabine led to a significant hypermethylation of the *MLH1* promoter as assessed by methylation-sensitive PCR (Fig. 6A). This increase in methylation was accompanied by reduced *MLH1* expression (Fig. 6B). In contrast, etoposide was without significant effect.

Epigenetic therapy is becoming an increasingly important strategy for cancer treatment since cancer cells show genome wide epigenetic alterations. For example, many tumor suppressor genes are hypermethylated while the bulk of the genome is hypomethylated [30], [31]. However, clinical drugs affecting DNA methylation are limited to 5-azacytidine (Vidaza) and its derivative 5-aza-2′-deoxycytidine (decitabine, Dacogen), both of which induce DNA hypomethylation (reviewed in [32]). Previously it was shown that a number of cytotoxic anticancer drugs which block DNA replication induce DNA hypermethylation. It was proposed that this effect is due to methylation of CpGs at stalled replication forks, which would normally not be methylated [33], [34]. However, the doses required in these experiments were in the micro- to millimolar range, and thus 1000x higher than the doses used in our experiments. Therefore the physiological- or clinical relevance of this “cytotoxic hypermethylation” effect is unclear. Unlike “cytotoxic hypermethylation”, gemcitabine did not affect global DNA methylation and did not markedly inhibit cell proliferation at the doses used in our experiments (data not shown).

Our results rather support a model where gemcitabine functions by inhibiting NER and thereby DNA demethylation, thus leading to gene silencing. We therefore propose that gemcitabine besides its various known effects also acts as an epigenetic drug on DNA methylation, which has consequences for the understanding of its effect in cancer therapy. For example, *MLH1* is a tumor suppressor and the fact that its expression is silenced by gemcitabine may be an undesirable effect in cancer treatment. More generally, gemcitabine may be a useful tool to specifically interfere with Gadd45 mediated DNA demethylation in biological processes ranging from embryonic gene activation to adult neurogenesis [19], [29].

## Materials and Methods

### Tissue culture and transfection

HEK293, HEK293T, MCF7 and RKO cells (ATCC numbers: CRL-1573, CRL-11268, HTB-22, CRL-2577, respectively) were grown at 37°C in 10% CO_2_ (5% CO_2_ for RKO cells) in Dulbecco's Modified Eagle's Medium (DMEM), 10% fetal calf serum, 2 mM L-Glutamine, 100 U/ml penicillin and 100 µg/ml streptomycin. HCT116 cells (ATCC number: CCL-247) were cultured at 37°C under 10% CO_2_ in McCoy's 5A medium supplemented as described above. Transient DNA transfections were carried out using FuGENE6 (Roche) following the manufacturer instructions. For MLH1 and C1S2 methylation analysis, cells were treated with 34, 67 or 134 nM gemcitabine (Eli Lilly) or 43 nM etoposide (Sigma Aldrich) for 18 h or with 500 nM 5-aza-2′-deoxycytidine (AZA, Sigma) for 42 h before harvesting. For methylation-sensitive Southern blotting and bisulfite sequencing, cells were transfected on 10 cm dishes with 1.2 µg *pBl-KS* control plasmid or *Gadd45a* along with *pOctTK-EGFP*. 3 h after transfection, cells were treated with 134 nM gemcitabine for 65 h. For methylation-sensitive PCR of *pOctTK-EGFP* at HpaII site −299, cells were transfected in 6-well dishes with 100 ng *pBl-KS* control plasmid or *hGadd45a* along with 200 ng *pOctTK-EGFP* using Turbofect transfection reagent (Fermentas) following the manufacturer instructions. Immediately after transfection, cells were treated with 50, 100 or 150 nM gemcitabine, 15, 25 or 50 nM camptothecin (MP Biomedicals), 50, 100 or 200 µM CRT 0044876 (Sigma Aldrich), 1, 5 or 10 µM betulinic acid (Sigma Aldrich), 5, 10 or 20 µM ABT-888 (Tebu Bio) or 10, 20 or 40 nM etoposide (Sigma Aldrich) for 48 h.

### Luciferase reporter assay

Dual-Luciferase reporter assays (Promega) were performed 40 h after transient DNA transfection of HEK293T cells in 96-well plates with a total of 110 ng DNA per well, containing 5 ng firefly luciferase reporter, 5 ng pBS or 5 ng *Xenopus tropicalis Gadd45a* plasmid, 0.1 ng *Renilla* luciferase reporter plasmid and 100 ng pBS. Reporter plasmids were produced in the *dam* ^−^/*dcm* ^−^ bacteria strain SCS110 and *in vitro* methylated using the *HpaII*- and *HhaI*-methylase. Transfections were performed in triplicate. Where indicated, cells were treated with 67 nM gemcitabine, 26 nM camptothecin (MP Biomedicals), 43 nM etoposide (Sigma-Aldrich), 30 nM β-lapachone (Calbiochem) or 20 nM merbarone (Calbiochem) for 18 h. Results are shown as the mean of triplicates and error bars indicate standard deviation. Experiments were repeated three times.

### Quantitative RT-PCR (qPCR)

RNA was isolated using the RNeasy Kit (Qiagen) and reverse transcribed with the SuperScript II reverse transcriptase (Invitrogen). Real-Time PCR was performed using Roche LightCycler480 probes master and primers in combination with predesigned mono-color hydrolysis probes of the Roche Universal probe library (UPL). The following primers and UPL probes were designed at https://www.roche-applied-science.com/sis/rtpcr/upl/adc.jsp. hMLH1 forward 5′- GAATGCGCTATGTTCTATTCCA, reverse 5′-ATGGAGCCAGGCACTTCA, UPL probe #38. For quantification Roche LC480 relative quantification software module was used. All values were normalized to the level of the housekeeping gene *GAPDH*.

### Analysis of DNA methylation

Genomic DNA (gnDNA) from treated cells or transfected reporter plasmids were prepared using the Blood & Tissue kit (Qiagen). The DNA was split into three parts and either digested with PvuII, HpaII or its methylation insensitive isoschizomer MspI. Methylation was determined by comparing HpaII digested versus PvuII control digested DNA samples via qPCR using methylation sensitive PCR primers (HpaII hMLH1 forward, 5′- CCTCAGCAGAGGCACACA; reverse, 5′- CGGGGAATACGAAATATCCA in combination with SYBR green; HpaII pOctTK -299 forward, 5′- ATAACCAGCCACCTTGATCTG; reverse, 5′- ATTCGCCAATGACAAGACG in combination with Roche UPL probe #39). As internal normalization control, a PCR using methylation insensitive primers (for gnDNA: forward, 5′-CTCCAACTCAGGGCCTACAC; reverse, 5′-CCAGGCTTTTGTGGCCTAT in combination with SYBR green; for *pOctTK* plasmid: forward, 5′-ACTGCATCTCCCTTTCCTTGT; reverse, 5′-GCCCCCTGCAAGTCTTTT in combination with Roche UPL probe #137) was performed (data not shown). MspI digest served as control for an intact restriction enzyme recognition site. To control for complete HpaII digest, amplification of the promoter of the unmethylated *GAPDH* housekeeping gene containing two HpaII sites (data not shown) or the unmethylated reporter plasmid was performed.

COBRA was performed as described [35]. Genomic DNA methylation levels were determined by capillary electrophoretic analysis, as described [36].

Methylation-sensitive Southern blotting was performed as described previously [13]. For bisulfite sequencing, the transfected *pOctTK-EGFP* reporter plasmid was recovered from the cells using alkaline lysis as described [37], subjected to another round of purification using the DNA MiniPrep Kit (Qiagen). The recovered plasmid DNA was linearized by NotI restriction digest and 500 ng DNA were bisulfite converted using the Epitect Kit (Qiagen). 2.5 µl of the converted DNA was used as template for PCR amplification using Accuprime Taq DNA polymerase (Invitrogen) and the following primers: forward, 5′ GATTTGTTTTGTAGGTGGAGAGTTT; reverse, AAATAAACTTCAAAATCAACTTACC. The PCR product was cloned using the TA cloning kit (Invitrogen) and single clones sent for sequencing. The experiment was reproduced three times with very similar results.

### BrdU incorporation in Xenopus oocytes

BrdU incorporation assays were performed essentially as described [13]. 5 fmol gemcitabine was injected with 5 pmol BrdU and 10 pg HpaII/HhaI *in vitro* methylated *oct4* plasmid. Plasmid DNA was recovered from oocytes harvested 0, 12 or 36 h after injection. BrdU-labeled control DNA *(BrdU-Luc)* generated by nick-translation was added during lysis to monitor the immunoprecipitation.

## Supporting Information

Figure S1 Full *in vitro* methylation of the *pOctTK-EGFP* reporter plasmid. Bisulfite sequencing analysis of five HpaII sites within the *pOctTK-EGFP* reporter upon *in vitro* methylation using HpaII methylase. The sequencing reveals that the plasmid used for transient transfection in Figure 1C, 2A and B was fully *in vitro* methylated. Therefore, changes upon transfection are indicative for endogenous DNA demethylation. White and black circles, unmethylated, methylated CpG, respectively. Arrow marks GFP translation start site.
(1.64 MB EPS)

Figure S2 *pOctTK-EGFP* reporter plasmid does not replicate during DNA demethylation. For these experiments, the transfected reporter plasmid was amplified using Dam methylase positive *E. coli* (dam+). The HpaII *in vitro* methylated reporter was then transiently transfected with or without *hGadd45a* in presence or absence of gemcitabine (Gem, 150 nM). 65 h after transfection the reporter was recovered for methylation sensitive PCR. Shown are the results of two independent experiments (exp. 1 and 2). Untransfected reporter plasmids that were either unmethylated (HpaII non-me) or HpaII *in vitro* methylated (dark grey bars) served as reference. They were either amplified in dam+ cells or in dam negative *E. coli* (dam-) as indicated. (A) HpaII methylation sensitive PCR. In agreement with Figure 3, the *in vitro* methylated CpG at position -299 is demethylated by *hGadd45a*. Note: the lower overall methylation level compared to Figure 3 is due to the longer incubation time of 65 h versus 48 h. As expected, the untransfected HpaII *in vitro* methylated plasmid is resistant to HpaII digest, whereas non-methylated is fully digested. (B) ClaI methylation sensitive PCR. A single ClaI recognition site (ATCGAT) in the backbone of *pOctTK* is also target for bacterial Dam methylation. Overlapping bacterial Dam methylation blocks ClaI restriction at this site. During replication in eukaryotic cells, the bacterial methylation would be diluted if the plasmid was replicated and would gain ClaI sensitivity. Accordingly, the untransfected reporter from dam- cells is sensitive to ClaI. However, the transfected *pOctTK* from dam+ *E. coli* remains as resistant to ClaI digest as the untransfected plasmid (dark grey bar, dam+). This is expected for a non-replicating plasmid.
(3.10 MB EPS)
